# Rewiring of mitochondrial metabolism in therapy-resistant cancers: permanent and plastic adaptations

**DOI:** 10.3389/fcell.2023.1254313

**Published:** 2023-09-13

**Authors:** Katherine E. Pendleton, Karen Wang, Gloria V. Echeverria

**Affiliations:** ^1^ Lester and Sue Smith Breast Center, Baylor College of Medicine, Houston, TX, United States; ^2^ Dan L. Duncan Cancer Center, Baylor College of Medicine, Houston, TX, United States; ^3^ Department of Medicine, Baylor College of Medicine, Houston, TX, United States; ^4^ Department of Molecular and Cellular Biology, Baylor College of Medicine, Houston, TX, United States; ^5^ Department of BioSciences, Rice University, Houston, TX, United States

**Keywords:** metabolism, cancer, resistance, plasticity, oxphos (oxidative phosphorylation)

## Abstract

Deregulation of tumor cell metabolism is widely recognized as a “hallmark of cancer.” Many of the selective pressures encountered by tumor cells, such as exposure to anticancer therapies, navigation of the metastatic cascade, and communication with the tumor microenvironment, can elicit further rewiring of tumor cell metabolism. Furthermore, phenotypic plasticity has been recently appreciated as an emerging “hallmark of cancer.” Mitochondria are dynamic organelles and central hubs of metabolism whose roles in cancers have been a major focus of numerous studies. Importantly, therapeutic approaches targeting mitochondria are being developed. Interestingly, both plastic (i.e., reversible) and permanent (i.e., stable) metabolic adaptations have been observed following exposure to anticancer therapeutics. Understanding the plastic or permanent nature of these mechanisms is of crucial importance for devising the initiation, duration, and sequential nature of metabolism-targeting therapies. In this review, we compare permanent and plastic mitochondrial mechanisms driving therapy resistance. We also discuss experimental models of therapy-induced metabolic adaptation, therapeutic implications for targeting permanent and plastic metabolic states, and clinical implications of metabolic adaptations. While the plasticity of metabolic adaptations can make effective therapeutic treatment challenging, understanding the mechanisms behind these plastic phenotypes may lead to promising clinical interventions that will ultimately lead to better overall care for cancer patients.

## 1 Introduction

During organismal development, cellular plasticity allows progenitor cells to differentiate into terminal states to produce specific tissues or functions (Tata and Rajagopal, 2016). Once cells are terminally differentiated, there is typically an inability to access previous cellular states. Increasing evidence suggests that cancer cells can hijack these mechanisms and access cellular states or metabolic functions that promote survival under environmental stressors. This can manifest in several ways: dedifferentiation of terminal states to progenitor states, blocked differentiation of progenitor cells, and trans-differentiation of progenitor cells into alternate differentiated states. Given its impact on promoting tumor progression, cellular plasticity is now regarded as a hallmark of cancer (Hanahan, 2022). Upon exposure to anticancer therapy, the innate heterogeneity of tumor cells can enable Darwinian selection of lineages that can withstand therapy. The refractory, residual tumor cells may then achieve a permanent (i.e., stable) adaptation enabling them to survive. This permanent resistance is often a result of evolution of *de novo* genomic alterations and/or clonal selection of pre-existing resistant subclones (Hanahan, 2022).

Alternatively, residual tumor cells may enter a plastic (i.e., reversible) state that can be reverted upon discontinuation of drug treatment (Boumahdi and de Sauvage, 2020). This is achieved through genetic (Balko et al., 2014; Rambow et al., 2018; Hong et al., 2019), epigenetic (Sharma et al., 2010; Roesch et al., 2013; Risom et al., 2018; Ravindran Menon et al., 2020), transcriptional (Menendez et al., 2004a; Vazquez-Martin et al., 2007a), and metabolic (Martinez-Outschoorn et al., 2014; Moldogazieva et al., 2020; Ravindran Menon et al., 2020) mechanisms to survive the onslaught of drugs. Individual or combinations of these mechanisms can sometimes produce evasion strategies that lead to a more stem-like residual population that is better poised to revert to alternative cellular states (Creighton et al., 2009; Hennessy et al., 2009; Rambow et al., 2018). Through an ongoing process of differentiation and dedifferentiation that is spearheaded by epigenetic alterations, tumor cells may revert between cancer stem cells (CSCs) and non-CSCs to evade anticancer therapy. The accumulation and loss of epigenetic alterations in response to anticancer therapy may also provide temporary, plastic adaptations beneficial for survival (Echeverria et al., 2019). Furthermore, plastic adaptations may only be present in the context of a drug. During a drug holiday, plastic phenotypic changes can revert to a drug-sensitive state (Gatenby et al., 2009; Gallaher et al., 2018). While there are many experimental models of permanent drug resistance, there are few that recapitulate the plastic properties of tumor cells that attain resistance and revert after drug discontinuation. These specialized models include patient derived xenografts (PDXs) (Echeverria et al., 2019; Baek et al., 2023), cell lines (Sharma et al., 2010), and genetically engineered mouse models (GEMMs) (Viale et al., 2014; Beerling et al., 2016). Distinguishing between plastic and permanent adaptations must be addressed so that rationally scheduled drug dosing regimens can be strategized to target residual cells that are refractory to standard-of-care therapies.

Recently, there is growing interest in metabolic-driven mechanisms of cancer resistance as well as the development of anti-cancer drugs that target the mitochondria. Indeed, metabolic plasticity has been found to be a common adaptation in many tumor types, promoting tumor progression and metastasis (Li et al., 2020a; Cheung et al., 2020; Herkenne et al., 2020). While many therapies do not specifically target the mitochondria, resistant cells are nonetheless metabolically reprogrammed due to yet unknown survival mechanisms. In this review, we highlight therapy-induced permanent and plastic mitochondrial adaptations in cancer with an emphasis on therapeutic strategies that can be implemented to overcome resistance of refractory, residual cells.

## 2 Overview of mitochondrial metabolism

**FIGURE 1:**
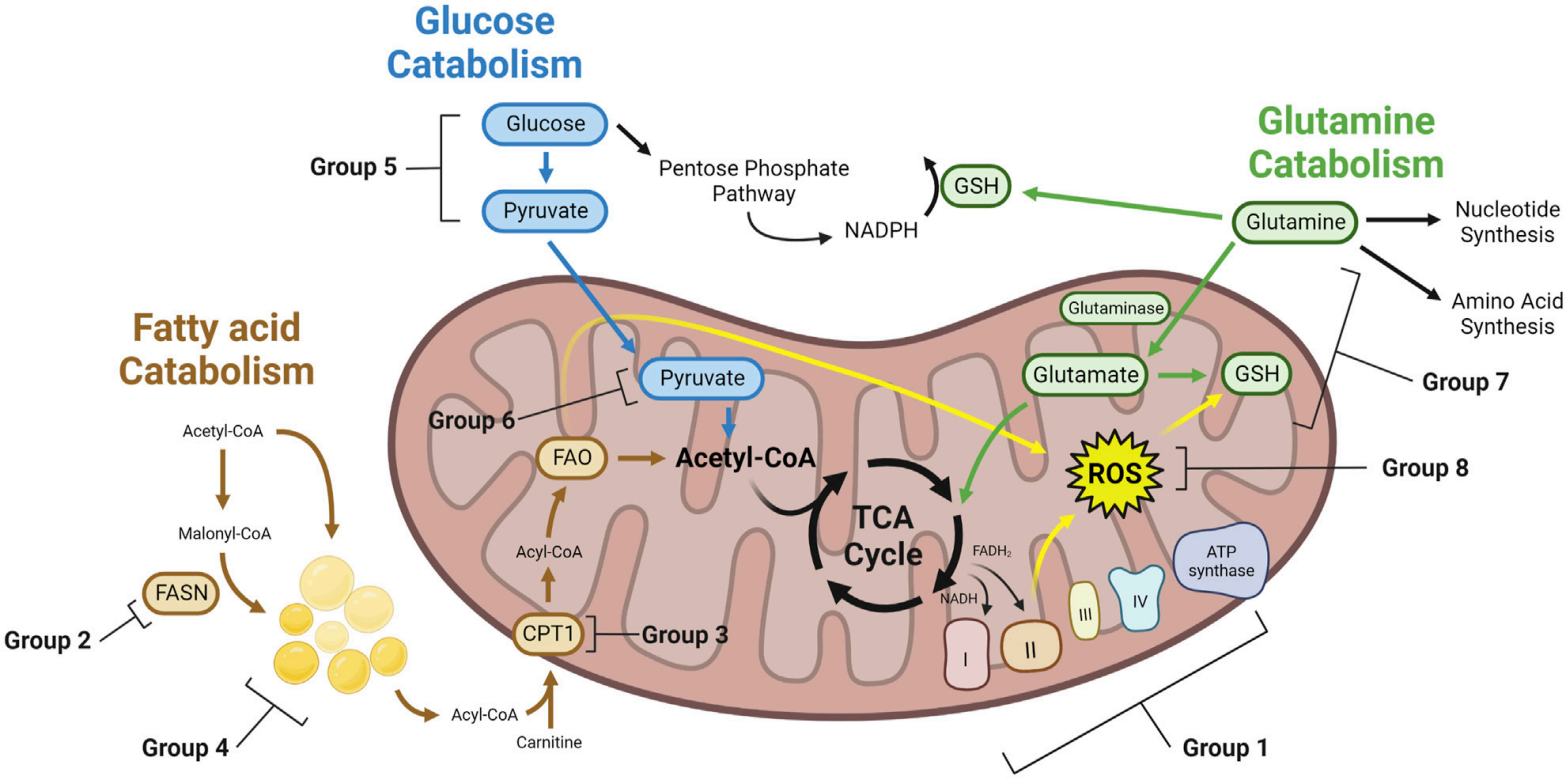
Metabolic mechanisms of therapy induced cancer resistance. Therapy resistant cancer cells with altered metabolism can achieve resistance through a variety of mechanisms. These cells may have greater TCA cycling, leading to increased NADH and FADH_2_ which feeds the ETC. To buffer ROS generated from the ETC, lipids, and other cellular reactions, therapy resistant cells may upregulate enzymes that consume ROS. GSH generated from glutamine can be a key enzyme to buffer ROS. Glucose can generate pyruvate through glycolysis, which can then enter the mitochondria and generate acetyl-CoA for the TCA cycle. Alternatively, glucose may feed the PPP. Similarly, fatty acids can be generated through FAS, forming LDs, or entering directly into the mitochondria to undergo FAO, providing acetyl-CoA for the TCA cycle. Glutamine can feed nucleotide synthesis, amino acid synthesis, or into the mitochondria and the TCA cycle as glutamate. Group numbers indicate the metabolic dependency of studies found in this paper, which can be found in Table 1.

Cellular energy metabolism includes two main pathways: cytoplasmic glycolysis and mitochondrial oxidative phosphorylation (oxphos). Glycolysis generates pyruvate and adenosine triphosphate (ATP) from glucose. This ancient process is highly conserved across species and allows for the rapid generation of two net ATP by converting pyruvate to lactate. Oxphos occurs in the mitochondria and produces large amounts of ATP by leveraging electron carriers, nicotinamide adenine dinucleotide (NADH) and flavin adenine dinucleotide (FADH_2_), produced in the tricarboxylic acid (TCA) cycle. The electrons from NADH and FADH_2_ then drive the electron transport chain (ETC) by passing through its complexes and ultimately generating ATP. During electron transport, leaky electrons can escape the ETC and interact with oxygen, generating reactive oxygen species (ROS). ROS act as secondary messengers and play a crucial role in regulating metabolic responses and homeostasis within and outside the mitochondria. Several energetic molecules can feed into the TCA cycle, including long chain fatty acids (LCFAs), glutamine, pyruvate, and nucleotides. LCFAs are generated in the cytoplasm during fatty acid synthesis (FAS) or are taken up from the extracellular space. LCFAs are then imported into the mitochondria through carnitine palmitoyl transferase 1 (CPT1) where they are broken down via fatty acid β-oxidation (FAO). The breakdown of the LCFAs produces acetyl-CoA, which can directly feed into the TCA cycle. Acetyl-CoA can also be generated from pyruvate that has been oxidized from glucose and imported into the mitochondria. Besides acetyl-CoA, cytoplasmic glutamine can provide an alternative source of carbons that feeds into the TCA cycle when it gets converted to α-ketoglutarate (AKG) (Figure 1). The TCA cycle is highly reversible and adaptive. While these energetic molecules can feed the TCA cycle, intermediates derived from these molecules can also be shuttled out of the TCA cycle to participate in other biological pathways. Examples include the citrate-malate shuttle of acetyl-CoA from the mitochondria to the cytoplasm to drive FAS or the conversion of oxaloacetate or malate to pyruvate. Several in depth reviews of lipid, glucose, and glutamine metabolism have been published recently (Hay, 2016; Choi and Park, 2018; Fernández et al., 2020; Fu et al., 2020; Lin et al., 2020; Yoo et al., 2020).

Tumors are complex ecosystems consisting of cancer cells, immune cells, endothelial cells, fibroblasts, and many others. These non-tumor stromal cells play a significant role in tumor resistance and survival (Bussard et al., 2016). In particular, many immune cells, such as cancer associated fibroblasts or tumor associated macrophages, have known pro-tumor properties (Ni et al., 2021). These pro-tumor immune cells may be metabolically reprogrammed to rely on oxphos, which provides an opportunity to target mitochondrial metabolism in both tumor and immune cells (Pearce and Pearce, 2013; Biswas, 2015). While the stroma plays a significant role in drug resistance and metabolic reprogramming, the focus of this review will be on therapy resistant, oxphos reliant tumor cells. Comprehensive reviews detailing metabolic reprogramming of stromal cells in cancer are available (Pearce and Pearce, 2013; Biswas, 2015; Bussard et al., 2016; Ni et al., 2021).

## 3 Therapy-induced rewiring of mitochondrial metabolism: permanent mechansims

Mitochondrial metabolism has been a target of interest in recent years for cancer treatment. Altered mitochondrial metabolism in cancer was first described by Otto Warburg with his finding that tumors relied on glycolysis rather than oxphos for their energetic needs (Warburg, 1925). It has since been well established that some cancer types including breast cancer (Lee et al., 2017; Echeverria et al., 2019; Baek et al., 2023), prostate cancer (Ippolito et al., 2016), melanoma (Haq et al., 2013), pancreatic cancer (Viale et al., 2014), leukemia (Kuntz et al., 2017), and glioblastoma (Janiszewska et al., 2012) rely on oxphos more so than on glycolysis in certain contexts. It is not clear why certain cancers rely more on oxphos than others. One possibility is that some tumors have higher amounts of mitochondrial DNA (mtDNA) compared to normal tissue, translating to increased oxphos capacity (Yu, 2011; Reznik et al., 2016). Further, some cancers may be enriched for metastatic CSCs, which have been demonstrated to rely on oxphos more so than non-CSCs in pancreatic cancer (Viale et al., 2014; Viale et al., 2015).

An ongoing challenge in cancer therapy is treating drug resistance in surviving cells. Reliance on mitochondrial metabolism in tumors cells presents a potential vulnerability that could be targeted via oxphos inhibitors to eliminate the residual surviving cells. Below, we first review instances of non-plastic oxphos rewiring in which therapeutics induce changes that do not appear to revert. Then, we cover therapy-induced changes in the TCA cycle and mitochondrial redox state that are correlated with resistance and/or residual cell survival. Lastly, we discuss how residual tumor cells can utilize different energy sources such as lipids, glucose, and glutamine to fuel oxphos, as well as oxphos inhibitors and inhibitors that target the production, import, or catabolism of these fuels. A summary of studies discussed in this review that demonstrate therapy induced metabolic rewiring and drug resistance can be found in Table 1.

**TABLE 1:** Metabolic dependencies of therapy resistant cancer cells. Studies in this paper that have identified metabolic alterations in therapy resistant cancers are listed. Group numbers indicate the mechanism of metabolic rewiring.

Group ID	Group dependency	References	First author	Cancer	Resistant to	Therapy to overcome metabolic resistance	Metabolic dependence mechanism	Evidence of oxphos alterations	Evidence for permanent or plastic oxphos adaptation
1	ETC	Lee et al. (2018)	Lee et al.	NSCLC	Irinotecan	Phenformin	Complex I of the ETC	Yes	Permanent
1	ETC	Ippolito et al. (2016)	Ippolito et al.	Prostate	Docetaxel	Metformin	Complex I of the ETC	Yes	Permanent
1	ETC	Farge et al. (2017)	Farge et al.	AML	Cytarabine	Metformin	Complex I of the ETC	Yes	Permanent
1	ETC	Wu et al. (2018)	Wu et al.	HCC	Doxorubicin	Rotenone	Complex I of the ETC	Yes	Permanent
1	ETC	McGuirk et al. (2021)	McGuirk et al.	Breast	Epirubicin	Biguanide phenformin	Complex I of the ETC	Yes	Permanent
1	ETC	Echeverria et al. (2019)	Echeverria et al.	TNBC, AML	Adriamycin Cyclophosphamide	IACS-010759	Complex I of the ETC	Yes	Plastic
1	ETC	Xu et al. (2022)	Xu et al.	Bladder	Cisplatin	Phenformin	Complex I of the ETC	Yes	Plastic
1	ETC	Krysztofiak et al. (2021)	Krysztofiak et al.	Lung, Liver, Colorectal, Prostate, Brain, Breast	Ionizing Radiation	Rotenone	Complex I of the ETC	Yes	Plastic
1	ETC	Wu et al. (2018)	Wu et al.	HCC	Doxorubicin	Oligomycin	Complex V of the ETC	Yes	Permanent
2	FAS	Vazquez-Martin et al. (2007a)	Vazquez-Martin et al.	Breast	Trastuzumab	C75	FASN	Untested	N/A
2	FAS	Menendez et al. (2004b)	Menendez et al.	Breast	Docetaxel	Cerulean	FASN	Untested	N/A
2	FAS	Menendez et al. (2005b)	Menendez et al.	Breast	Paclitaxel	C75	FASN	Untested	N/A
2	FAS	Liu et al. (2008)	Liu et al.	Breast	Adriamycin	Orlistat	FASN	Untested	N/A
2	FAS	Vazquez-Martin et al. (2007b)	Vazquez-Martin et al.	Breast	5-FU	Cerulenin	FASN	Untested	N/A
2	FAS	Menendez et al. (2004c)	Menendez et al.	Breast	Vinorelbine	Cerulenin	FASN	Untested	N/A
3	FAO	Tan et al. (2022)	Tan et al.	Ovarian	Cisplatin	Etomoxir	CPT1	Yes	Permanent
3	FAO	Huang et al. (2021)	Huang et al.	Ovarian	Carboplatin	Etomoxir or Perhexiline	CPT1	Yes	Permanent
3	FAO	Salunkhe et al. (2020)	Salunkhe et al.	Leukemia	Mitoxantrone	Etomoxir	CPT1	Yes	Permanent
3	FAO	Li et al. (2022)	Li et al.	Breast	Paclitaxel	Knockdown of STAT3 and ACSL4	CPT1	Yes	Permanent
3	FAO	Wang et al. (2018)	Wang et al.	Breast	Paclitaxel	Perhexiline	CPT1	Yes	Permanent
3	FAO	Stevens et al. (2020)	Stevens et al.	AML	Venetoclax and Azacytidine	Etomoxir	CPT1	Yes	Permanent
3	FAO	Farge et al. (2017)	Farge et al.	AML	Cytarabine	Etomoxir	CPT1	Yes	Permanent
4	LDs	Cotte et al. (2018)	Cotte et al.	Colorectal	Oxaliplatin and 5-FU	TSI-01	LPCAT2	Untested	N/A
4	LDs	Huang et al. (2019)	Huang et al.	NSCLC	Gefitinib	g-PPT	SCD1	Untested	N/A
4	LDs	Sirois et al. (2019)	Sirois et al.	Breast	Doxorubicin or Paclitaxel	GW9662	PPARG/PLIN4	Yes	Permanent
4	LDs	Chen et al. (2020)	Chen et al.	Ovarian	Cisplatin	LLC	ABCs	Untested	N/A
5	Glycolysis	Shi et al. (2019)	Shi et al.	Colorectal	Oxaliplatin and 5-FU	2-DG	HK2	Untested	N/A
5	Glycolysis	He and Liu (2020)	He et al.	NSCLC	Cisplatin	3-Bromopyruvate Acid	HK2	Untested	N/A
5	Glycolysis	Qian et al. (2017)	Qian et al.	Gastric	Cisplatin	2-DG	ENO1	Untested	N/A
5	Glycolysis	Deng et al. (2021)	Deng et al.	Colorectal	5-FU	miR-488	PFKFB3	Untested	N/a
5	Glycolysis	He et al. (2014)	He et al.	Colorectal	5-FU	miR-122	PKM2	Untested	N/A
5	Glycolysis	Pan et al. (2016)	Pan et al.	Liver	Doxorubicin	miR-122	PKM2	Untested	N/A
5	Glycolysis	Zhou et al. (2010)	Zhou et al.	Breast	Paclitaxel	Oxamate	LDH	Untested	N/A
5	Glycolysis	Xu et al. (2018)	Xu et al.	Ovarian	Cisplatin	ABT737, 2-DG	Bcl-2, G6PD	Yes	Permanent
5	Glycolysis	Catanzaro et al. (2015)	Catanzaro et al.	Ovarian	Cisplatin	6-aminonicotinamide	G6PDH	Yes	Permanent
5	Glycolysis	Zub et al. (2015)	Zub et al.	MM	Melphalan alkylating chemotherapy	dichloroacetate (DCA)	Glycolysis	Untested	N/A
5	Glycolysis	Zhou et al. (2015)	Zhou et al.	Lung	Paclitaxel	dichloroacetate (DCA)	Glycolysis	Untested	N/A
6	Glucose Oxidation	Marx et al. (2022)	Marx et al.	Colorectal	Irinotecan	Glucose deprivation	not tested	Yes	Permanent
6	Glucose Oxidation	Baek et al. (2023)	Baek et al.	Breast	Doxorubicin or Carboplatin	not tested	not tested	Yes	Plastic
6	Glucose Oxidation	Cai et al. (2020)	Cai et al.	Liver	Cisplatin	Metformin	GLUT1	Untested	N/A
7	Glutamine Metabolism	McGuirk et al. (2021)	McGuirk et al.	Breast	Doxorubicin	Buthionine sulfoximine	Glutamate-cysteine ligase	Yes	Permanent
7	Glutamine Metabolism	Thompson et al. (2017)	Thompson	MM	Carfilzomib	CB-839	Glutaminase (GLS)	Yes	Permanent
7	Glutamine Metabolism	Catanzaro et al. (2015)	Catanzaro et al.	Ovarian	Cisplatin	Glutamine free media	GLUT1	Yes	Permanent
7	Glutamine Metabolism	Hernandez-Davies et al. (2015)	Hernandez-Davies et al.	Melanoma	Vemurafenib	BPTES and L-DON	Glutaminase (GLS)	Untested	N/a
7	Glutamine Metabolism	Catanzaro et al. (2015)	Catanzaro et al.	Ovarian and Cervical	Cisplatin	2-DG	GLUT1	Yes	Permanent
7	Glutamine Metabolism	Hudson et al. (2016)	Hudson et al.	Ovarian	Cisplatin	BPTES	Glutaminase (GLS)	Yes	Permanent
7	Glutamine Metabolism	Baenke et al. (2016)	Baenke et al.	Melanoma	PLX4720	BPTES	Glutaminase (GLS)	Yes	Permanent
7	Glutamine Metabolism	van Gastel et al. (2020)	van Gastel et al.	AML	Cytarabine and Doxorubicin	L-DON	Glutamine metabolism	Untested	N/A
8	ROS	Song et al. (2020)	Song et al.	Breast	Gefitinib	Knockdown of GPX4	GPX4	Untested	N/A
8	ROS	Corazao-Rozas et al. (2013)	Corazao-Rozas et al.	Melanoma	PLX420	Elesclomol	PGC1α	Yes	Permanent
8	ROS	Lee et al. (2018)	Lee et al.	NSCLC	Irinotecan	Gossypol	ALDH	Yes	Permanent
8	ROS	Lee et al. (2020)	Lee et al.	Colon, melanoma, prostate, ovary, stomach, pancreatic	Irinotecan or Vemurafenib or Cisplatin or Doxorubicin	Gossypol	ALDH	Yes	Permanent
8	ROS	Xu et al. (2018)	Xu et al.	Ovarian	Cisplatin	ABT737, 2-DG	Bcl-2, G6PD	Yes	Permanent
9	Other	Pandit et al. (2021)	Pandit et al.	HCC	Sorafenib	OPB-111077	STAT	Yes	Permanent
9	Other	Bosc et al. (2021)	Bosc et al.	AML	Cytarabine	Venetoclax	BCL-2	Yes	Permanent
9	Other	Farge et al. (2017)	Farge et al.	AML	Cytarabine	Tigecycline	Mitochondrial protein translation	Yes	Permanent
9	Other	Aroua et al. (2020)	Aroua et al.	AML	Cytarabine	Polyoxometalate 1	ENTPD1	Yes	Permanent
9	Other	van Gastel et al. (2020)	van Gastel et al.	AML	Cytarabine and Doxorubicin	Brequinar	Pyrimidine Synthesis	Untested	N/A

Many studies have demonstrated an enhanced reliance on mitochondrial oxphos following the stress of therapeutic exposure in various cancer types. In one study, irinotecan resistant non-small cell lung cancer (NSCLC) cells had increased oxphos compared to treatment-naïve NSCLC cells (Lee et al., 2018). When the resistant cells were targeted with gossypol, an inhibitor of aldehyde dehydrogenase (ALDH), and phenformin, an inhibitor of Complex I of the ETC, cells were sensitized to irinotecan (Lee et al., 2018). It has been shown that cancer cells can rely more on cytosolic NADH production via ALDH whereas normal cells rely more on the TCA cycle for NADH production (Kim, 2018; Kim, 2019; Lee et al., 2019). Therefore, by targeting ALDH via gossypol in combination with anticancer drugs (irinotecan, vemurafenib, cisplatin, and doxorubicin), anticancer effects were increased in mouse xenograft cancers such as colon, melanoma, ovary, prostate, stomach, and pancreatic (Lee et al., 2020). Oncogenic pathway targeting has also been shown to result in pro-oxphos adaptations (Kantarjian et al., 2002; Flaherty et al., 2010). For example, in pancreatic ductal adenocarcinoma (PDAC), tumor cells that survived Kirsten rat sarcoma viral oncogene homolog (KRAS) oncogene ablation were reliant on oxphos and had features similar to cancer stem cells (Viale et al., 2014). In turn, these surviving cells were highly sensitive to oxphos inhibitors (Viale et al., 2014). Furthermore, hepatocellular carcinoma cells (HCC) resistant to sorafenib, a tyrosine kinase inhibitor, had increased proteins necessary for mitochondrial protein translation and import, suggestive of increased mitochondrial metabolism (Pandit et al., 2021). Because phosphorylated signal transducer and activator of transcription (STAT) 3 (pSer727) was also increased in the resistant HCC cells, treatment with a small-molecule STAT inhibitor reverted the phosphorylation and subsequent mitochondrial protein alterations, enhancing sensitivity to sorafenib (Pandit et al., 2021).

Resistance to conventional chemotherapies remains a major clinical challenge. Several cancers exhibit increased oxphos in chemoresistant cells, and in some cases, this presents a therapeutic vulnerability. Docetaxel resistant prostate cells exhibit a reliance on oxphos through the utilization of glucose, glutamine, and lactate (Ippolito et al., 2016). By inhibiting mitochondrial Complex I via metformin in docetaxel resistant PC3 prostate cells, proliferation and invasiveness were impaired (Ippolito et al., 2016). This effect was only seen in the resistant cells but not in parental cells, suggesting that Complex I is an effective target in prostate cells that have achieved oxphos reliance after chemotherapy (Ippolito et al., 2016). In hepatocellular carcinoma, doxorubicin resistant cells had increased reliance on the oxphos pathway while the sensitive cells were more reliant on glycolysis (Wu et al., 2018). Additionally, when the doxorubicin resistant cells were treated with rotenone and oligomycin, inhibitors of Complex I and Complex V the ETC, respectively, ATP levels were significantly reduced, suggesting that oxphos was critical in the energy production of the resistant cells and that it could present a potential therapeutic target (Wu et al., 2018). In breast cancer, epirubicin resistant cells had increased mitochondrial ATP production which was shown to be a vulnerability upon treatment with biguanide phenformin, an inhibitor of Complex I of the ETC, in cell lines and tumor xenografts (McGuirk et al., 2021). In acute myeloid leukemia (AML), primary cancer cells that were resistant to cytarabine (AraC) and had a high “MitoScore,” which identified cells with high reliance on oxphos, were highly reliant on B cell lymphoma-2 (BCL-2) expression (Bosc et al., 2021). When treated with venetoclax, an inhibitor of BCL-2, and AraC, the AML cells were sensitive. Furthermore, when AraC-resistant AML cells were treated with venetoclax and inhibitors of the ETC, there was delayed relapse (Bosc et al., 2021). Reliance on oxphos through BCL-2 regulation in chemoresistant AML has been shown elsewhere as well (Lagadinou et al., 2013). In line with the finding that chemoresistant AML cells were more reliant on oxphos, another study found that residual AML PDX cells resistant to AraC had increased mitochondrial mass, high levels of ROS, and gene signatures high in oxphos (Farge et al., 2017). Subsequent treatments with drugs targeting FAO, mitochondrial protein synthesis, and the ETC in combination with AraC enhanced cell killing vs. AraC alone in the chemoresistant models (Farge et al., 2017). In another study, ectonucleoside triphosphate diphosphohydrolase-1 (ENTPD1) was upregulated in AraC resistant AML which was associated with enhanced mitochondrial activity (Aroua et al., 2020). When ENTPD1 was inhibited in the AraC resistant AML patient cells, mitochondrial reprogramming was blocked and the killing effects of AraC were enhanced (Aroua et al., 2020). In all three cases of AML AraC resistance, oxphos was a therapeutic vulnerability that could be targeted. However, in other tumor types, reduced oxphos is an adaptation in chemoresistant cells. For example, cisplatin-resistant ovarian cells had reduced mitochondrial activity, reduced oxygen consumption, and were more reliant on glycolysis and the pentose phosphate pathway (PPP) (Catanzaro et al., 2015). There was increased expression of the PPP enzyme Glucose-6-Phosphate Dehydrogenase (G6PDH). The authors found that the resistant ovarian cells were more sensitive when G6PDH was inhibited (Catanzaro et al., 2015). Together, these studies show that oxphos can be modulated by cytotoxic chemotherapies as well as pathway-targeted therapies and that the nature and directionality of the oxphos rewiring is highly cancer type-dependent.

### 3.1 Therapy-induced adaptations in TCA cycle proteins and metabolites

Although Otto Warburg found that cancer cells may rely on glycolysis rather than oxphos for their energetic needs, studies in more recent years have found that certain cancers may preferentially utilize oxphos rather than glycolysis. Oxphos is the primary process through which cells generate ATP, one that requires the usage of electron carriers such as NADH and FADH_2_, produced mainly in the TCA cycle. Recent studies are shedding light on the roles that alteration in the TCA cycle enzymes can play in oxphos dependence and in turn, therapy resistance in cancers.

Some studies show an increased contribution towards TCA cycle metabolites correlated with drug-resistance. Metabolomic profiling of gastric cancer cells made resistant to the anti-HER2 monoclonal antibody trastuzumab revealed that resistant cell lines maintained heightened levels of citrate synthase (CS) and ATP citrate lyase (ACLY), and had a significant increase in the metabolites citrate, fumarate, and phosphoenolpyruvic acid, compared to their sensitive counterparts (Liu et al., 2019). Given that ACLY cleaves citrate into oxaloacetate and acetyl-CoA, the increase in citrate and ACLY levels induced by trastuzumab could reflect: 1) a need for TCA cycle fueled energy through oxphos as well as 2) an alteration of lipid metabolism in trastuzumab resistant cells. Additionally, ACLY plays an important role in epigenetic regulation, as acetyl-CoA is a major source of acetyl marks in histone acetylation. While not investigated in the study, trastuzumab resistant cells could also harbor epigenetic modifications that further alter the metabolic profile of resistant cells.

In another study, an estrogen receptor positive breast cancer cell line made resistant to doxorubicin favored anaplerotic (metabolism involved in replenishing oxaloacetate, a crucial intermediate of the TCA cycle) pyruvate metabolism. Doxorubicin resistant cells showed significantly increased RNA expression of anaplerotic genes pyruvate carboxylase, malic enzyme 1 (ME1), and malic enzyme 2 (ME2), corroborated by the enrichment of TCA cycle intermediates citrate, malate, and fumarate (Kim, 2018). Other studies indicate a more direct link between upregulated TCA cycle flux and increased oxphos activity in drug-resistant cells. In cytarabine resistant acute myeloid leukemia (AML) cells, increased TCA cycle intermediates such as citrate, AKG, succinate, fumarate, and malate were associated with increased oxphos compared to sensitive cells (Farge et al., 2017). Interestingly, this was not correlated with significant changes in pyruvate and glucose consumption, or glutamine consumption, suggesting the resistant cells were taking up oxidizable substrates for the TCA cycle from other sources such as amino acids or fatty acids (Farge et al., 2017).

Some studies also indicate that there does not necessarily need to be a shift away from glycolysis for there to be increased TCA cycle activity and oxphos (Chong et al., 2022). In a study of doxorubicin resistant breast cancer cells, levels of glycolytic intermediates such as fructose-6-phosphate and pyruvate were increased, as well as TCA cycle intermediates citrate, isocitrate, AKG, fumarate, and malate (Chong et al., 2022). The study showed that decreased glucose concentrations in the resistant cells resulted from increased activity in both glycolytic pathways and the TCA cycle. A similar phenotype was observed in doxorubicin resistant hepatocellular carcinoma, where both oxphos and glycolysis were upregulated (Wu et al., 2018). In this study, the authors found that the resistant cell line possessed a great dependency on AKG: suppression of AKG metabolism and transfer through the mitochondria greatly reduced the of viability drug-resistant tumor cells (Wu et al., 2018). In residual triple-negative breast cancer (TNBC) cells following chemotherapy treatment (with either DNA-damaging agents or taxanes), both glucose and lactate flux into the TCA cycle were observed to be increased (Baek et al., 2023). However, only DNA-damaging chemotherapies increased oxphos, whereas taxane chemotherapies decreased oxphos. Together, these studies show that oxphos can be modulated by cytotoxic chemotherapies as well as pathway-targeted therapies and that the nature and directionality of the oxphos rewiring is highly cancer type-dependent. The reason(s) for this are currently unclear.

### 3.2 Therapy-induced adaptations in mitochondrial redox state

Reduction-oxidation (redox) chemical reactions involve the transfer of an electron between a reducing agent (electron donor) and an oxidizing agent (electron acceptor) (Ahmad et al., 2023). The movement of electrons between species is necessary for energy production and many cellular processes. Within the mitochondrial matrix, redox reactions comprise most TCA cycle reactions, generating the reducing cofactors NADH and FADH_2_. These high-energy electron carriers (reducing agents) provide the electrons to fuel the ETC embedded in the inner mitochondrial membrane. NADH donates an electron at Complex I, generating NAD^+^, while FADH_2_ donates two electrons at Complex II, generating FAD. Electrons are then passed through the ETC complexes, providing the energy required to move hydrogen atoms across the inner mitochondrial membrane into the intermembrane space, generating an electrochemical proton gradient. Electrons are ultimately donated to molecular oxygen by Complex IV to form water. Lastly, ATP synthase (Complex V) generates ATP from ADP by passing protons from the intermembrane space (Ahmad et al., 2023). Therefore, mitochondrial redox reactions are crucial steps in ATP generation and cellular homeostasis. An in-depth overview of redox reactions within the cell and mitochondria has been previously published (Ghoneum et al., 2020). Herein we explore therapy-induced alterations in mitochondrial redox state associated with oxphos adaptations and implications in cancer.

Oxphos reliance in drug resistant cancers comes with the cost of generating reactive oxygen species (ROS). ROS are generated when electrons “escape” their intended oxidizing agent and bind oxygen molecules to form radicals. Therefore, increased ETC activity naturally leads to increased ROS production. In addition to oxphos, major sources of ROS include NADPH oxidases and to a lesser extent peroxidases, cyclooxygenases, cytochrome p450, and xanthine oxidase (de Almeida et al., 2022). At high levels, ROS can have a variety of toxic effects within cells, such as damage to DNA and proteins. To account for this, some drug resistant cancers with high oxphos have developed mechanisms to buffer elevated ROS. ALDHs are enzymes that scavenge ROS through their ability to metabolize aldehydes (Singh et al., 2013). In fact, targeting ALDH can successfully decrease ATP production in cancer (Kang et al., 2016a; Kang et al., 2016b; Lee et al., 2018). For example, irinotecan-resistant pancreatic and gastric cancer cell lines were found to have increased oxphos. Treatment with gossypol, an inhibitor of ALDH, reduced oxphos in the resistant lines *in vitro* while the combination of gossypol, irinotecan, and phenformin (Complex I inhibitor) synergistically reduced tumor size in therapy naive cells *in vivo* (Lee et al., 2020). Therefore, higher ALDH expression may predispose tumors to resistance. Higher oxphos and ALDH expression were noted in gastric cancer cell lines resistant to crizotinib, a tyrosine kinase inhibitor (Raha et al., 2014). Additionally, the combination of disulfiram, an ALDH inhibitor, with erlotinib, an EGFR inhibitor, significantly delayed tumor regrowth in lung cancer xenografts. Importantly, disulfiram treatment continued until the end of the experiment. This may suggest that erlotinib reprogrammed surviving cells towards oxphos reliance, making them more vulnerable to ALDH inhibition compared to the monotherapies (Raha et al., 2014). Head and neck (HNC) cancer cell lines resistant to the PI3K inhibitor BEZ235 showed increased ROS and ALDH expression. It is possible that the increased ROS was buffered, in part by ALDH, however there was no functional assessment of oxphos levels in this study to understand the source of the ROS (Hsueh et al., 2021). Instead, the authors noted that superoxide dismutase 2 (SOD2) was increased in the resistant cells. The superoxide dismutase (SOD) family of proteins catalyze the generation of H_2_O_2_, a stable ROS species, from highly reactive superoxide O_2_^−^ molecules. By reducing O_2_^−^, SOD proteins limit cell damage caused by this unstable radical (Ghoneum et al., 2020). Further, SOD2 is in the mitochondrial matrix and is the major processor of ETC generated superoxide (Karnati et al., 2013). This may suggest that ROS generated within the mitochondria of the BEZ235 resistant cells was due to oxphos activity (Hsueh et al., 2021). Sirtuins (SIRTs), a family of deacetylases, may be able to buffer oxphos generated ROS in cancer (Kim et al., 2010; Bell et al., 2011). SIRT1 is a histone deacetylase that requires NAD^+^ and is implicated in mitochondrial biogenesis (Li and Laher, 2014). Human colon cancers that had been treated with many types of chemotherapy exhibited a SIRT1-dependent oxphos increase. Knock down of SIRT1 in combination with 5-FU in colon cancer xenografts reduced oxphos and increased sensitivity to chemotherapy (Vellinga et al., 2015). These findings could indicate that oxphos-generated ROS is buffered by SIRT1 in chemotherapy resistant colon cancer.

Outside the mitochondria, redox reactions are used heavily in glucose, glutamine, and lipid metabolism. Glucose and glutamine generate NADPH while glutamine also generates glutathione (GSH), which is made through a series of redox reactions (Alberghina and Gaglio, 2014; Ghoneum et al., 2020). GSH is an antioxidant and redox buffer and is often necessary for tumor cell survival (DeBerardinis and Cheng, 2010). Greater oxphos, ROS, superoxide, and glucose import were noted in cisplatin resistant ovarian cancer cells (Xu et al., 2018). The authors also noted greater expression of G6PD, the rate-limiting enzyme of the PPP, in resistant cells. Increased activity of the PPP can generate more GSH to buffer ROS. Indeed, by inhibiting BCL-2 through ABT737 and glycolysis through 2-DG, GSH and glucose metabolism-related proteins were reduced more so in resistant lines compared to sensitive, suggesting the glucose mediated ROS was a vulnerability in the chemoresistant cells (Xu et al., 2018). Increased GSH and glutamine incorporation into the TCA cycle was also noted in another study of cisplatin resistant ovarian cancer (Catanzaro et al., 2015).

Lipid redox reactions also produce ROS and can induce a unique type of cell death called ferroptosis (Wang et al., 2021). Iron accumulates to counteract increased lipid ROS, generating lipid peroxides that induce ferroptosis. These lipid peroxides can be counteracted by glutathione peroxidase 4 (GPX4), an enzyme that catalyzes GSH into oxidized GSH and reduces lipid peroxides to alcohols (Li et al., 2020b). Gefitinib resistant TNBC cells had greater expression of GPX4 compared to sensitive cells, and when GPX4 was knocked down, the cells were more sensitive to gefitinib (Song et al., 2020). By silencing GPX4, GSH decreased, allowing greater ROS accumulation, and ferroptosis was no longer inhibited, which facilitated cell death in the resistant lines. Silencing GPX4 in combination with gefitinib treatment reduced tumor growth and decreased ROS in tumor xenografts. While metabolism was not explicitly investigated in this study, decreased mitochondrial membrane potential was observed in GPX4 knock down cells (Song et al., 2020). This may indicate that oxphos could be contributing to the altered ROS and redox state in these models. Further, inhibitors of GPX4 are currently being developed to induce ferroptosis, suggesting that targeting this pathway may be feasible in cancer (Liu et al., 2022; Randolph et al., 2023). In contrast, FAO mediated ROS could be an adaptation in drug resistant cells. Using a transgenic mouse model of HER2 driven breast cancer, the authors could increase or decrease HER2 expression with doxycycline (Fox et al., 2020). Tumors with reduced HER2 were more vulnerable to treatment with etomoxir (FAO inhibitor) and accumulated less ROS, suggesting that loss of HER2 induces an adaptation towards greater FAO and mitochondrial ROS. This was supported by greater expression of FAO related genes, such as the acylcarnitine transporters and CPT1A and CPT1B, and greater lipid droplet (LD) accumulation in mammospheres with HER2 inhibition (Fox et al., 2020). The concept of increased FAO in drug resistant cells as a method to maintain ROS levels has been documented in several other studies as well (Aloia et al., 2019; Shen et al., 2020; You et al., 2021).

Sometimes, decreased ROS levels may be adaptive within drug resistant cells, even if their oxphos levels are high. Gastric cancer cell lines were subjected to chronic metabolic stress through prolonged culturing in nutrient deprived media to generate stem-like cancer cells (Choi et al., 2020). CSCs are noted for their drug resistant properties, which can include a metabolic adaptation towards oxphos utilization (Phi et al., 2018). In the gastric stem like cancer cells, oxphos was increased, but paradoxically, ROS were decreased compared to non-CSCs. A reduction in ROS was found to be mediated by peroxiredoxin 3 (PRX3), which was increased in the CSCs. PRX3 is a mitochondrial antioxidant enzyme which can reduce ROS. Further, NADPH, which can serve as the reducing agent for PRX3, was increased in the CSCs, and the source of NADPH was linked to FAO (Choi et al., 2020). In line with these findings, a ROS-low phenotype in drug resistant leukemia CSCs has been noted. The increased oxphos but low ROS in primary leukemia stem cells was controlled by BCL-2, which was transcriptionally upregulated in these cells and a regulator of oxphos. Inhibition of BCL-2 with ABT-263 resulted in decreased oxphos and increased ROS, which induced cell death (Lagadinou et al., 2013). While these studies are not direct models of drug resistance, they suggest that an adaptation of cells that survive cytotoxic stress, such as chemotherapy, may increase oxphos while upregulating proteins that can buffer mitochondrial ROS.

In summary, when drug resistant cells upregulate oxphos, electrons produced from redox reactions within the ETC result in increased ROS which can have toxic effects if not mediated. Cancer cells have a variety of mechanisms to buffer ROS and identifying these mechanisms may present targetable vulnerabilities in drug resistant tumors.

### 3.3 Therapy-induced adaptations of oxphos via lipid metabolism

Fatty acids are high-energy molecules whose production is fueled by the TCA cycle intermediate acetyl-CoA and whose break-down can fuel TCA cycling and oxphos (Figure 1). Altered fatty acid metabolism in cancer cells has been implicated in the pathogenesis of many cancer types (Koundouros and Poulogiannis, 2020). Fatty acid metabolism is an attractive therapeutic target, as there are many promising inhibitors that target fatty FAS or FAO in the mitochondria such as etomoxir (phase II clinical trials for treatment of congestive heart failure), orlistat (FDA approved treatment for weight loss), omeprazole (FDA approved treatment for heartburn and other conditions), and others.

Fatty acid synthase (FASN) is the rate-limiting enzyme for lipogenesis of LCFAs in the cytosol. Several studies have demonstrated a role for FASN in the resistance of HER2 over-expressing breast cancers to standard of care agents including cytotoxic chemotherapies and anti-HER2 therapies (Ligorio et al., 2021). Blocking FASN via orlistat or siRNA knockdown was found to inhibit transcription of HER2 by upregulating PEA3, a transcriptional repressor of HER2, although the molecular mechanism is not yet understood (Menendez et al., 2005a). Trastuzumab, an anti-HER2 antibody, is therefore thought to abrogate FASN-induced upregulation of HER2 via PEA3. In recognition of this vulnerability, combination with a FASN inhibitor and trastuzumab sensitized trastuzumab-resistant breast cancer cell lines by downregulating HER2 expression (Vazquez-Martin et al., 2007a). A later study further investigated the relationship between FASN and HER2 signaling, revealing that HER2 directly phosphorylates FASN when dimerized, resulting in increased enzymatic activity of FASN (Jin et al., 2010). FASN activity was also found to be crucial for survival of taxane-resistant HER2-over-expressing breast cancer cells. This revealed synergism of the FASN inhibitor cerulean with docetaxel in HER2 over-expressing breast cancer cell line models (Menendez et al., 2004b). Inhibiting FASN in cell lines of several breast cancer subtypes have effectively re-sensitized the cells to treatment with conventional chemotherapies paclitaxel (Menendez et al., 2005b), Adriamycin (Liu et al., 2008), 5-FU (Vazquez-Martin et al., 2007b), and vinorelbine (Menendez et al., 2004c). Together, these results indicate that some breast cancers may have increased resistance to therapy given their reliance on FAS. HER2 is also a driver of gastric (Gravalos and Jimeno, 2008; Begnami et al., 2011), bladder (Eissa et al., 2005), and non-small cell lung cancers (Nakamura et al., 2003; Liu et al., 2010). High HER2 and FASN expression are associated with tumor recurrence and progression in bladder cancer, however the mechanistic details of HER2-FASN interactions have not been explored (Abdelrahman et al., 2019). Further studies are needed to assess if the HER2-FASN regulatory axis is relevant in other cancers.

Targeting FAO has proved promising as well. Resistance to cisplatin is a crucial determinant of overall survival in high grade serous ovarian cancer. Several studies have demonstrated the importance of FAO in residual ovarian cancer cells that are refractory to cisplatin. In one study, continuous treatment of ovarian cancer cell lines with cisplatin led to higher fatty acid uptake and subsequent FAO in the resistant cells (Tan et al., 2022). Cisplatin resistant cells had higher oxphos than nonresistant cells, and upon treatment with etomoxir had a larger decrease in oxphos compared to nonresistant cells. The resistant cells were also more sensitive to cisplatin when co-treated with etomoxir, suggesting the cisplatin resistant cells may be more reliant on FAO to drive oxphos and that this reliance is a therapeutic vulnerability. In line with these findings, oxphos and FA metabolic gene expression pathways were increased in carboplatin resistant ovarian cancer cell lines and PDXs compared to sensitive (Huang et al., 2021). Furthermore, CPT1A expression was associated with carboplatin resistance in the cell lines. Treatment with CPT1 inhibitors etomoxir or perhexiline sensitized the ovarian cancer cell lines to carboplatin. Preventing LCFA import into the mitochondria has shown efficacy in mitoxantrone-resistant HL-60 leukemia cells. These resistant cells had increased LDs and oxphos compared to sensitive cells (Salunkhe et al., 2020). The addition of etomoxir to the resistant cells decreased colony formation and the addition of an oxphos inhibitor, Antimycin-A, increased sensitivity of the resistant cells to mitoxantrone (Salunkhe et al., 2020).

The JAK/STAT pathway may play an important role in upregulating CPT1 transcription to promote chemoresistance. In paclitaxel resistant TNBC cell lines, increased acetyl-CoA due to FAO was found to acetylate STAT3 (Li et al., 2022). Acetylated STAT3 binds to the promoters of CPT1B and long-chain fatty acid coa ligase 4 (ACSL4), key proteins involved in FAO and FAS, respectively. Inducible knockdown of STAT3 and ACSL4 in a paclitaxel resistant TNBC cell line decreased tumor growth and membrane lipids *in vivo*, suggesting that acetylated-STAT3 promotes FAO-mediated chemoresistance. A similar finding was documented in another study in which CTP1B and STAT3 were higher in post chemotherapy TNBC patient tumors compared to pre-chemotherapy biopsies (Wang et al., 2018). Paclitaxel resistant cell lines also showed a greater decrease in viability when treated with paclitaxel and perhexiline compared to the sensitive controls (Wang et al., 2018). FAO appears to regulate epithelial to mesenchymal transition (EMT) in paclitaxel resistant breast cancer cells. By broadly inhibiting FAO genes using bexarotene, a type of retinoid, mammosphere forming ability was disrupted (Stevens et al., 2020). Further, venetoclax and azacytidine (ven/aza) resistant AML cells had upregulated oxphos which was driven by upregulated FAO. FAO in the resistant cells was revealed to be a targetable vulnerability when FAO was inhibited by siRNAs against FA genes and etomoxir, resulting in increased sensitivity to ven/aza (Stevens et al., 2020). Upregulated FAO gene signatures were also seen in AraC resistant AML cells by single-cell transcriptomics (Bosc et al., 2021).

Rates of FAO and FAS can be intrinsically tied to LDs, which are organelles containing neutral lipids encapsulated by a phospholipid monolayer. Originally considered an energy storage structure, LDs are now appreciated to play a dynamic role in lipid metabolism and energy homeostasis. LDs first emerge from the endoplasmic reticulum using neutral lipids generated from FAS and can associate with many organelles in the cell, such as the mitochondria, for a variety of functions. LDs are a well-documented phenotype of cancer cells (Petan, 2023). They can sequester lipophilic drugs, reducing their efficacy (Zhang et al., 2016; Cotte et al., 2018). LDs that contact mitochondria are poised to provide highly energetic fatty acids to the mitochondria to promote FAO when glucose sources are low (Rambold et al., 2015; Nguyen et al., 2017). As such, LDs play a major role in anti-cancer drug resistance. LD accumulation has been associated with therapy resistance across cancer types through various mechanisms. For example, LD accumulation was increased in colorectal cancer cells resistant to oxaliplatin and 5-FU both *in vitro* and *in vivo* (Cotte et al., 2018). Further, the LDs helped mediate resistance to cell death by preventing ER-mediated stress responses and caspase activation (Cotte et al., 2018). In NSCLC cell lines and patient tissues, tyrosine kinase inhibitor (gefitinib) resistant cells had more LDs compared to their sensitive controls with greater expression of stearoyl-CoA desaturase 1 (SCD1) (Huang et al., 2019). SCD1 is an enzyme that generates oleic acid and other monounsaturated fatty acids. Inhibiting SCD1 with 20(S)-protopanaxatriol (g-PPT) decreased SCD1 expression in the NSCLC cells, resulting in decreased LD content (Huang et al., 2019).

Increased LD accumulation in drug resistant cells can be a result of increased lipid uptake rather than *de novo* lipogenesis. Indeed, cisplatin resistant ovarian cancer cell lines had increased LDs which were not abrogated by the addition of a FASN inhibitor, C-75, suggesting that FAS was not responsible for the increased LDs seen in the resistant cells (Tan et al., 2022). Chemotherapy itself may be causing increased LDs in surviving, resistant cells. In TNBC, cell lines dosed with a single, high dose of either doxorubicin or paclitaxel resulted in a greater accumulation of LDs, as well as heightened FAO, in the surviving cells (Sirois et al., 2019). Within tumors and chemoresistant cell lines, a dependency on perilipin 4 (PLIN4), a protein that coats LDs, was noted (Sirois et al., 2019). It is possible the cells that survive chemotherapy are enriched for polyaneuploid cancer cells (PACCs), which have been found to have a greater proportion of LDs compared to non-PACC cancer cells (Kostecka et al., 2021). PACCs are large, multinucleated cells that are thought to help mediate resistance and metastasis and are generated usually by a stressor, such as chemotherapy or hypoxia (Illidge et al., 2000; Mittal et al., 2017; Mirzayans et al., 2018; Amend et al., 2019). A greater proportion of PACCs and LDs, therefore, could mediate survival by sequestering lipophilic drugs to prevent cytotoxicity. The Lands Cycle, which reversibly converts an acyl group between phosphatidylcholine (PC) and lysophosphatidylcholine (LPC), mediates the generation of LDs. In a computational analysis of epithelial ovarian carcinoma (EOC) cells, the cells that survived cisplatin did so in part through extracellular disposal of cisplatin through ATP-binding cassette genes (ABCs) from LDs, avoiding cytotoxic effects of the drug (Chen et al., 2020). The export of cisplatin could be due to a defective Lands Cycle which preferentially generates more PC than LPC. This was supported by a lipidomic analysis of cancer cell lines treated with a lysophosphatidylcholine liposome cisplatin drug (LLC). The LLC resistant lines were reprogrammed towards greater LPC generation. This, in turn, promoted the cellular transport of cisplatin to DNA, increasing the amount of DNA-platinum adducts and cell death (Chen et al., 2020). Reprogramming the Lands Cycle has shown utility in KRAS-mutant lung cancer as well (Bartolacci et al., 2022). Lung cancer cells were found to utilize the Lands Cycle to reduce polyunsaturated FAs (PUFAs) and phospholipids, which can induce ferroptosis. Targeting the Lands Cycle through lysophosphatidylcholine acyltransferase 3 (LPCAT3) or phospholipase A2 group IVC (PLA2G4C) inhibition resulted in increased ferroptosis, indicating that proper functioning of the Lands Cycle is necessary to prevent cell death in KRAS-mutant lung cancer (Bartolacci et al., 2022). In summary, FAS, FAO, exogenous lipid uptake, and FA storage are all mechanisms by which tumor cells can rewire metabolism to evade therapy. In some instances, targeting these mechanisms has shown promise to overcome drug resistance.

### 3.4 Therapy-induced adaptations of oxphos via glucose metabolism

Traditionally, glucose metabolism in the context of cancer has been focused on the canonical “Warburg Effect,” which postulates that cancer cells preferentially utilize glucose for the production of lactate during aerobic glycolysis, thus generating energy quickly even in cells with defective mitochondria (Warburg, 1925). However, a growing body of research indicates that tumor cells can readily convert glucose into pyruvate which can be shuttled into the mitochondria to drive the TCA cycle and oxphos (Pavlides et al., 2009). This “Reverse Warburg” effect, as well as the canonical Warburg effect, have been documented in drug resistant cells, presenting an opportunity to target glucose catabolism in drug resistant cancers. Examples of glucose metabolism rewiring in therapy resistant cancers are numerous and have been recently reviewed (Liu et al., 2021). We discuss a select few studies below exemplifying some of the mechanisms by which cancer therapies can impinge upon glycolysis.

Given that many cancers upregulate glycolysis, targeting enzymes involved in glucose catabolism is under investigation for overcoming therapy resistance. Several studies have demonstrated direct modulation of glycolysis enzyme levels in therapy-resistant tumor cells. B7-H3, an immunoregulatory protein, was found to confer resistance to oxaliplatin and 5-FU in colorectal cancer cell lines through upregulation of hexokinase 2 (HK2) leading to increased glucose and lactate consumption (Shi et al., 2019). Xenografted colorectal cancer cells overexpressing B7-H3 were more resistant to oxaliplatin while treatment with 2-Deoxy-D-glucose (2-DG), an HK2 inhibitor, reduced tumor size and tumor regrowth rate. Furthermore, patient tissues also had greater HK2 expression in the cisplatin resistant tumors compared to sensitive (Shi et al., 2019). This was supported in another study, where cisplatin resistant NSCLC cells also had increased glucose uptake *in vitro* and *in vivo* compared to sensitive NSCLC cells, which could be abrogated through HK2 inhibition (He and Liu, 2020). In another study, glycolytic enzyme enolase 1 (ENO1), and in turn glycolysis, were increased in cisplatin resistant gastric cancer cells, which could be targeted by the glucose analog 2-DG (Qian et al., 2017). ENO1 knockdown in the cisplatin resistant cells resulted in increased sensitivity to cisplatin, indicating glycolytic dependence was regulated by this enzyme. The oncogenic potential of ENO1 was supported in human gastric cancer tissues, in which higher ENO1 expression associated with shorter overall survival (Qian et al., 2017). Targeting other glycolytic enzymes has proved beneficial in additional chemoresistant cancers: 6-phosphofructo-2-kinase/fructose-2,6-biphosphatase 3 (PFKFB3) targeting by miR-488 reduced glucose uptake and increased sensitivity to 5-FU in colorectal cancer (Deng et al., 2021), PKM2 targeting by miR-122 decreased glycolysis and led to increased sensitivity to 5-FU in colorectal cancer (He et al., 2014) and in doxorubicin resistant liver cancer (Pan et al., 2016), and LDH targeting by siRNA knockdown or an inhibitor increased sensitivity in paclitaxel resistant breast cancer and showed synergy in combination with paclitaxel (Zhou et al., 2010). In a study on radioresistant cervical cancer cell lines, the authors found that resistant cells were more sensitive to glycolytic inhibition by 2-DG than radiosensitive cells (Rashmi et al., 2018). Melphalan alkylating chemotherapy is a common treatment for multiple myeloma (MM) and achieves a 70%–80% response rate but faces the issue of drug resistance and relapse. Pathway analysis of a melphalan resistant MM cell line revealed that glycolytic enzymes were upregulated while TCA cycle and ETC proteins were downregulated (Zub et al., 2015). Moreover, inhibiting glycolysis by treating resistant cells with 2-DG had a significantly stronger inhibitory effect on the growth of resistant cells compared to that of sensitive cells (Zub et al., 2015). Interestingly, dichloroacetate (DCA), an inhibitor that does not directly target glycolytic enzymes, mediated the largest selective effect against the resistant cells. DCA inhibits all pyruvate dehydrogenase kinase isoenzymes, thus increasing the activity of pyruvate dehydrogenase and increasing the amount of acetyl-CoA entering the TCA cycle. While DCA does not directly target the glycolytic pathway, the study observed that DCA treatment selectively induced mitochondrial ROS in the melphalan-resistant cells, which disrupted the mitochondrial ability to regenerate NAD^+^ to support glycolytic ATP-production (Zub et al., 2015). In a different study, dichloroacetate restored sensitivity to paclitaxel in lung carcinoma cells by inducing citric acid accumulation, inhibiting glycolysis through the inhibition of phosphofructokinase activity (Zhou et al., 2015)**.** Glycolysis as a therapeutic target has been well established modality for overcoming therapy induced resistance in preclinical models, however, depending on anaerobic or aerobic conditions, lactate or pyruvate can be the product, respectively. Pyruvate can then be imported into the mitochondria and undergo oxidation. Therefore, given the increasing evidence that mitochondrial metabolism is upregulated in cancers, there is likelihood that glycolysis driven cancers may also contribute to pyruvate mediated mitochondrial oxphos. This presents a potential vulnerability that could be targeted in addition to the well-established glycolysis targeting strategy.

Next, we explore studies which provide evidence that glucose-fueled oxphos is an adaptation in therapy resistant cancers. Reprogramming of glucose metabolism has been observed in pancreatic tumor cell lines made resistant to the standard of care nucleoside analog gemcitabine. While sensitive cells used glucose for aerobic glycolysis, resistant cells funneled glucose into nucleotide biosynthesis (Gebregiworgis et al., 2018). Further supporting the notion of glucose dependent metabolic switching after drug treatment, p53^+^ colorectal cells surviving treatment with irinotecan, a topoisomerase-1 inhibitor, had increased oxphos (Marx et al., 2022). Upon glucose restriction, the p53^+^ cells were more sensitive to irinotecan as indicated by increased mitochondrial depolarization and cell death (Marx et al., 2022). In TNBC, oxphos was increased in cell lines and a PDX surviving DNA-damaging chemotherapy (Baek et al., 2023). To understand the source of the increased oxphos, heavy labeled isotope tracing of glucose was conducted, and it was evident that TNBC cells treated with a variety of chemotherapies had increased glucose tracing into TCA cycle intermediates compared to untreated cells (Baek et al., 2023). While these studies did not reveal the mechanism of the increased glucose derived oxphos, they provided evidence that mitochondrial glucose metabolism presents a metabolic vulnerability in therapy resistant cells.

In other cases, glucose import can be altered in therapy-resistant cells. Cisplatin-resistant ovarian cancer cells displayed reduced oxphos and increased dependency on glucose compared to their cisplatin-sensitive counterpart (Catanzaro et al., 2015). This was attributed to glucose transporter 1 (GLUT1) upregulation in resistant cells, and treatment with 2-DG resulted in more cell death than in the sensitive cells. Similar findings were obtained in a human cisplatin-resistant cervix squamous cancer cell line (Pandit et al., 2021). In contrast, metabolism in docetaxel resistant prostate cancer cells was shifted from a Warburg phenotype to a more oxphos-reliant phenotype when compared to that of docetaxel sensitive cells (PC3), involving increased utilization of glucose, glutamine, and lactate by oxphos (Ippolito et al., 2016). Resistant cells increased glucose and lactate oxidation to carbon dioxide through oxphos, while decreasing GLUT1 expression and thereby glucose uptake. Increased pyruvate promoted oxphos, whereas lactate production remained unchanged. Furthermore, genes associated with oxphos, such as pyruvate dehydrogenase E1 subunit alpha 1 (PDHA1), monocarboxylate transporter 1 (MCT1), dihydrolipoamide dehydrogenase (DLD), and MYC, were upregulated, while those associated with glycolysis were downregulated. Targeting mitochondrial Complex I with metformin selectively inhibited growth and invasiveness in the docetaxel resistant cells, suggesting the resistant cells’ dependency on oxphos as a resistant adaptation (Pearce and Pearce, 2013).

While increased glycolysis and glucose import, or hyperglycemia, is generally regarded as survival adaptation in tumors, there are instances where it may confer greater sensitivity to therapy (Pandey et al., 2011). Metformin was found to increase glucose uptake and GLUT1 expression in cisplatin resistant liver cancer cells, resulting in increased cytotoxicity (Cai et al., 2020). Increased glycolysis reduced expression of nuclear factor erythroid-2 related factor 2 (NRF2) in the resistant cells, a transcription factor that induces the oxidative stress response. In human liver cancer tissues, NRF2 protein expression was increased in the patients who received cisplatin compared to those who did not, suggesting NRF2 expression may be a potential biomarker for response to metformin (Cai et al., 2020). Sometimes, decreased protein expression may predict response to therapy. Bladder cancer patients who had low expression of retinoic acid-related protein (RORC) by IHC also had increased glycolysis and PPP intermediates compared to those with high RORC expression (Cao et al., 2019). Enhanced expression of RORC suppressed cell proliferation and glucose metabolism while increasing cisplatin induced apoptosis *in vivo* and *in vitro*. RORC binds the promoter region of PD-L1 and negatively regulates its expression, preventing the interaction of PD-L1 with integrin β6 (ITGB6), an upstream regulator of the focal adhesion kinase (FAK) signaling pathway. With decreased RORC, this pathway was more active, resulting in increased glucose import, glycolysis, and ultimately resistance (Cao et al., 2019).

### 3.5 Therapy-induced adaptations of oxphos via glutamine metabolism

Glutamine, the most abundant nonessential amino acid, is a multifunctional metabolite, driving synthesis of glutathione, amino acids, and nucleic acids, as well as serving as a fuel source for the TCA cycle. Glutamine import into the mitochondria via glutamine transporters enables glutaminase-mediated conversion to glutamate, which is converted to AKG by glutamate dehydrogenase. Some cancer cells preferentially depend on glutamine rather than other carbon sources to drive mitochondrial metabolism, making glutamine metabolism an attractive therapeutic target (Wang et al., 2020; Jin et al., 2023).

Metabolomic analysis of an estrogen receptor positive breast cancer cell line made resistant to doxorubicin revealed heightened incorporation of glutamine into the TCA cycle compared to that observed in sensitive cells (McGuirk et al., 2021). Doxorubicin resistant cells also exhibited significantly increased expression of glutamine metabolism genes (Kim, 2018). A similar phenomenon was observed in the epirubicin-resistant estrogen receptor positive cell line: increased oxphos was accompanied by the increased expression of glutamate metabolism genes under the transcriptional regulation of peroxisome proliferator-activated receptor gamma (PPARG) coactivator 1 alpha (PGC-1α) (McGuirk et al., 2021). In contrast, metabolomic analysis of two human TNBC cell lines revealed that rather than glutamine incorporation into the TCA cycle was unaltered upon chemotherapy exposure (Baek et al., 2023). These studies highlight that each tumor type may have its own unique metabolic adaptations.

Protease inhibitors are common treatments for MM, so navigating drug resistance to these agents is of clinical importance. MM cells made resistant to the proteasome inhibitor, carfilzomib, were found to have increased reliance on glutamine metabolism compared to sensitive cells (Thompson et al., 2017). To target this metabolic switch, the authors introduced CB-839, a glutaminase-1 inhibitor, to the proteasome resistant MM cells and discovered greater cytotoxicity in these cells than in sensitive cells (Thompson et al., 2017). CB-839 was investigated in a phase II clinical trial comparing the addition of CB-839 to cabozantinib in metastatic renal cell carcinoma, however, there was no improvement to progression free survival in the patients who received CB-839 (Tannir et al., 2022). It is important to note that the patient population was not biomarker-selected. Clinical trials that select patients more likely to respond to glutamine inhibitors are needed to further assess the efficacy of targeting glutamine metabolism in cancer. Additionally, it is possible that cabozantinib, an inhibitor of tyrosine kinases, is not the optimal drug to use in combination with CB-839.

BRAF inhibitors are commonly used for melanoma patients whose tumors have BRAF mutations; however, most patients eventually acquire drug resistance. Melanoma cells resistant to BRAF inhibitors have been shown to display increased oxphos (Corazao-Rozas et al., 2013; Baenke et al., 2016). In one study, two BRAF mutant melanoma cell lines that were made resistant to BRAF inhibitor PLX4720 exhibited increased dependency on oxphos (Baenke et al., 2016). PLX4720 resistant cells were found to be more sensitive to glutamine starvation than parental cells, suggesting a dependency on glutamine metabolism for the increased oxphos mediating resistance. Inhibiting glutaminase, an enzyme that hydrolyzes glutamine to glutamate, blocked PLX4720-resistant cell growth in both *in vitro* and *in vivo* models. Providing dimethyl α-ketoglutarate, a membrane permeable analogue of α-ketoglutarate, a downstream product of glutamate, rescued resistant cells (Baenke et al., 2016). In another study of melanoma resistant to a BRAF inhibitor, vemurafenib, resistant cells were more sensitive to glutaminase inhibitors than were their sensitive counterparts both *in vitro* and *in vivo* (Hernandez-Davies et al., 2015).

In some cases, a decrease in glucose metabolism can be compensated via glutamine. In a study on cisplatin-resistant lung cancer cells, resistant cells expressed lower levels of glycolytic proteins, produced significantly less lactate, and were more sensitive to a glycolysis inhibitor in hypoxic conditions (Wangpaichitr et al., 2017). Instead, cisplatin-resistant cells required glutamine for oxphos and primarily depended on oxphos as their source of energy. A cisplatin-resistant ovarian cancer cell line displayed reduced oxphos and increased dependency on glucose compared to the cisplatin-sensitive counterpart (Catanzaro et al., 2015). This was attributed to GLUT1 upregulation in resistant cells, and treatment with 2-DG resulted in more cell death than the cisplatin-sensitive cells. Similar findings were obtained in a human cisplatin-resistant cervix squamous cancer cell line (Catanzaro et al., 2015). However, while glucose utilization for oxphos was decreased, there was instead increased incorporation of glutamine-derived carbons into TCA cycle intermediates such as succinate, fumarate, and malate in resistant cells. Furthermore, when cultured in glutamine-free media, resistant cells exhibited a marked decrease in cell proliferation (Catanzaro et al., 2015). Glutamine reliance in cisplatin resistant ovarian cancer cell lines was also documented in another study (Hudson et al., 2016). Since cisplatin is standard-of-care for ovarian cancer, further investigation into cisplatin induced glutamine reliance could prove promising for overcoming cisplatin resistance in this cancer. In a study of hepatocellular carcinoma cells made resistant to doxorubicin, glutamine-driven production of ATP was found to be crucial for chemoresistance by fueling the ATP-demanding process of doxorubicin efflux through P-glycoprotein (Lee et al., 2021). Deprivation of glutamine, but not of glucose or fatty acids, was able to re-sensitize the chemoresistant cell line to doxorubicin treatment. The success of preclinical inhibition of glutamine metabolism may lie in the innate ability of glutamine to inhibit the toxic effects of ROS induced by DNA-damaging agents (Gwangwa et al., 2019; Hwang et al., 2021). Indeed, glutamine is a known mediator of oxidative stress mitigation through the production of glutathione (Yoo et al., 2020).

While glutamine plays an important role in mediating cellular ROS, is important to note that there are conditions where decreased glutamine metabolism predisposes tumors to therapy resistance and survival. Within the core of tumors, often there exists a hypoxic environment where cells are starved for essential nutrients, such as glutamine (Lin et al., 2017; Ahmadiankia et al., 2019). CSCs that are deprived of glutamine are thought to be the cell population contributing to drug resistance in tumor cores (Prasad et al., 2021). When glutamine was inhibited, an ovarian and colorectal cell line relied more on glycolysis while maintaining oxphos levels and had more CSC-like properties, suggesting that cells primed towards nutrient deprivation may have strategies to overcome cytotoxicity of glutamine inhibitors (Prasad et al., 2021). The ability to utilize glycolysis rather than glutamine under hypoxic conditions results in enhanced metabolic flexibility. Breast cancer cells were able to survive the harsh conditions of hypoxia by upregulating a glycolytic enzyme, 6-phosphofructo-2-kinase/fructose-2,6-biphosphatase-4 (PFKFB4), compared to the normoxic controls *in vitro* and *in vivo* (Dai et al., 2022). PFKFB4 upregulation also produced increased NADPH through the PPP, a reducing equivalent that can buffer hypoxia-generated ROS (Dai et al., 2022). Therefore, to successfully target glutamine metabolism, more studies are needed to assess what types of tumor conditions promote killing when glutamine is restricted. While drugs can promote metabolic rewiring that induces a reliance on glutamine, there exists metabolic heterogeneity even within tumors from the same tissue origin. However, given glutamine’s important role in controlling toxic ROS levels, glutamine inhibitors could be an emerging therapeutic strategy in combination with chemotherapeutics.

## 4 Therapy-induced rewiring of mitochondrial metabolism: evidence for plasticity

Plasticity is defined by a cell’s ability to adapt its phenotypes in response to the environment in a reversible manner through non-permanent molecular mechanisms, usually entailing epigenetic, transcriptional, post-transcriptional, translational, and/or metabolic rewiring. The plastic persister cell state was first described in PC9, an EGFR mutated NSCLC cell line (Sharma et al., 2010). When treated with erlotinib (an EGFR inhibitor), only 0.3% of the starting cells survived. This population was dubbed “drug tolerant persisters (DTPs)” (Sharma et al., 2010). Upon removal of the drug, colonies that grew from the DTPs were again sensitive to the EGFR inhibitor, showcasing the innate ability of tumor cells to temporarily adapt to the onslaught of a drug such that the entire population is not eradicated (Sharma et al., 2010). Since this seminal discovery, the presence of plastic, persister populations has been affirmed in many different cancer types treated with a variety of drugs: an additional study of NSCLC treated with an EGFR inhibitor (Hata et al., 2016), breast cancer treated with HER2 tyrosine kinase inhibitors (Chang et al., 2022), colon cancer treated with chemotherapy (Rehman et al., 2021), breast and prostate cell lines treated with many chemotherapies (Dhimolea et al., 2021), TNBC treated with chemotherapies (Echeverria et al., 2019; Baek et al., 2023), and melanoma treated with a BRAF inhibitor (Shaffer et al., 2017). Most DTPs remain arrested under the presence of a drug, but a small proportion can still achieve proliferation by maintaining cell cycle capacity (Oren et al., 2021). These cycling persisters are not genetically unique but are rather poised to harness adaptive programs more effectively than non-DTPs. Persisters are known to have a unique upregulation of antioxidant and FAO gene pathways compared to non-cycling persisters, which is thought to aid in their proliferative capacity (Roesch et al., 2013; Oren et al., 2021). On the other hand, while lipid metabolism pathways may be elevated in cancers, there can exist plasticity which makes targeting these pathways challenging. This was noted in a variety of cancer cell lines which utilized the sapienate biosynthesis pathway in addition to the canonical fatty acid desaturation pathway (Vriens et al., 2019). Both pathways had to be targeted to reduce proliferation *in vitro* and *in vivo*, highlighting the plastic utilization of these lipid desaturation programs (Vriens et al., 2019). It can be challenging to treat these plastic DTPs, but by understanding the unique vulnerabilities these cells utilize, such as a reliance on estrogen (Garon et al., 2013) and ferroptosis (Hangauer et al., 2017; Friedmann Angeli et al., 2019), effective interventions may be possible when rationally sequenced and combined with conventional standard-of-care therapies. While significant strides have been achieved in understanding and targeting plastic DTPs, alterations in mitochondrial metabolism in these plastic cells are poorly understood.

Reversible, plastic resistance in prostate, breast, colon, pancreatic, and non-small cell lung cancer cell line models has been shown to be a consequence of nutrient deprivation which introduces ROS and low cell proliferation, priming cells to adapt to anti-tumor agents such as the mitochondrial ETC Complex I inhibitor metformin (Marini et al., 2016; White et al., 2020; Kadonosono et al., 2022). To capitalize on this adaptation, cancers such as colon or breast could be treated with metabolic inhibitors to prevent oxphos or glycolysis mediated adaptations (Marini et al., 2016). However, caution must be considered when using metformin due to its many molecular targets, making precise inhibition of oxphos challenging (Rena et al., 2017).

Cancers have been shown to upregulate oxphos in a plastic manner which reverts upon removal of the drug. For example, an *in vivo* study of PDX models of TNBC revealed upregulated oxphos after treatment with conventional front-line combination chemotherapies in the drug-tolerant residual tumor cells compared to untreated tumor cells (Echeverria et al., 2019; Baek et al., 2023). This oxphos-high phenotype in the residual PDX cells reverted upon drug holiday, and when ETC Complex I was inhibited with IACS-010759, there was significant and specific decrease in the tumor regrowth rate from the residual state (Molina et al., 2018; Echeverria et al., 2019; Baek et al., 2023). IACS-010759 progressed to phase I clinical trials for AML but was terminated early due to toxicities (Yap et al., 2023). While oxphos inhibitors have been successful in the preclinical setting, further work is needed to test more selective inhibitors of oxphos for clinical translation. Indeed, phosphorylated acetyl-CoA carboxylase 1 (ACC1-pS79) and FASN, proteins involved in FA metabolism, were increased in the residual TNBC PDX compared to the untreated and relapsed states (Echeverria et al., 2019). It is possible that an inhibitor of FAS or FAO could be effective in this model and perhaps less toxic if translated to humans. Similarly, a plastic, rapid shift towards oxphos reliance was observed in bladder cancer after cisplatin treatment. Bladder cancer cell lines were tracked through fluorescence lifetime microscopy (FLIM) to measure the cells’ proportion of free vs. bound NAD(P)H redox states (Xu et al., 2022). Cells with an oxphos FLIM signature were enriched after cisplatin treatment. Further, glutamine tracing revealed a significant contribution of glutamine to the TCA cycle in the oxphos enriched cells. The addition of phenformin, a Complex I inhibitor, caused the oxphos reliant cells to switch to a glycolysis signature, highlighting the plastic nature of the oxphos reliant cells. Combination cisplatin and phenformin synergistically reduced cell survival, but no studies were conducted to assess if phenformin would have greater effect in the cisplatin residual setting (Xu et al., 2022). One of the hallmarks of cancer metabolic plasticity is a switch towards glycolysis even in the presence of oxygen. Inhibiting the ability of cancer cells to switch to glycolysis even if it results in enhanced oxphos function could be a therapeutic strategy in select contexts. In one study, TNBC cells were cultured long-term with a Complex I inhibitor causing an increase in mitochondrial function and a decrease in lactate, indicating a reduced ability to switch to glycolysis (Xu et al., 2015). Even though oxphos function was increased in the Complex I inhibitor resistant cells, they had reduced tumor growth compared to the parental lines. This reveals that targeting glycolysis rather than oxphos may be a better strategy in some cancers to prevent drug induced metabolic plasticity (Xu et al., 2015). Other studies indicate that it is possible to force an energetic switch in cancer cell metabolism from glycolysis to oxphos, potentially creating avenues for therapeutic intervention. Similar to the metabolic switch documented in TNBC PDX models treated with combination chemotherapy, just a single dose of ionizing radiation in luminal A breast, colon, and glioblastoma cancer cell lines caused an increase in ATP production and oxphos while decreasing lactate production (Lu et al., 2015). This process was regulated by mTOR relocalization to the mitochondria in response to irradiation of the cell lines. By inhibiting mTOR, the energetic switch from aerobic glycolysis to oxphos did not occur, resulting in decreased clonogenic survival (Lu et al., 2015).

The innate energetic plasticity of cancer cells could be a result of their tendency to attain a “hybrid phenotype” of both aerobic glycolysis and oxphos simultaneously, allowing them to readily transition between states given the environmental stress (Lehuédé et al., 2016; Yu et al., 2017; Desbats et al., 2020). Therefore, understanding if an anti-tumor agent pushes the energetic axis preferentially towards one state or the other is expected to be informative for therapeutic intervention. In some cases, both oxphos and aerobic glycolysis inhibitors may be ideal, as in the case of metformin combined with 2-DG to target both pathways successfully in multiple preclinical models *in vivo* and *in vitro* (Cheong et al., 2011). In another study, a single dose of ionizing radiation (IR) induced a plastic drop in both oxphos and glycolysis across a variety of cancer cell lines (Krysztofiak et al., 2021). Over time, the glycolytic capacity recovered in just 6 h while the oxphos capacity partially recovered after 24 h (Krysztofiak et al., 2021). By targeting the cells with both IR and inhibitors against oxphos and glycolysis, mitochondrial recovery and DNA repair was greatly slowed, suggesting that the plastic response to IR could be leveraged pharmacologically (Krysztofiak et al., 2021). With combinatorial metabolic-targeted agents, it will be imperative that harm to normal tissues is minimized for successful translation to the clinic.

A plastic reliance on glutamine metabolism and pyrimidine synthesis was noted in AML. Mouse models of AML were injected with mouse leukemic bone marrow cells, then treated with the combination treatment of cytarabine and doxorubicin (van Gastel et al., 2020). The residual, post chemotherapy cells had a distinct metabolic profile compared to the vehicle or relapsed mice that indicated a reliance on glutamine metabolism. Interestingly, inhibiting glutamine metabolism with 6-diazo-5-oxo-L-norleucine only improved survival when it was given in the residual setting, rather than in combination with the chemotherapy. This provided evidence that the plastic metabolic rewiring of the AML cells surviving chemotherapy provided a unique window for therapeutic targeting. Glutamine tracing revealed that glutamine was not feeding the TCA cycle but was rather driving the pyrimidine synthesis pathway. This was contrary to *in vitro* studies which indicate glutamine in AML does support the TCA cycle (Matre et al., 2016; Gregory et al., 2019). Timed inhibition of the pyrimidine synthesis pathway with brequinar in the residual chemotherapy setting improved survival in AML mouse models and PDXs, revealing that both glutamine and pyrimidine metabolism was a therapeutic vulnerability in the post chemotherapy setting (van Gastel et al., 2020). While this study did not provide strong evidence of oxphos reliance, it suggests that cells surviving drugs may have short term metabolic adaptive responses. Gaining understanding of the timing of these metabolic adaptations is critical for the sequential addition of metabolic drugs used in combination with standard-of-care therapy. A valuable lesson learned from longitudinal studies such as these is that the timing of metabolism-targeted interventions relative to standard-of-care treatments is of crucial importance. For this reason, preclinical studies elucidating not only the mechanistic, but also the temporal, nature of metabolic rewiring are essential.

## 5 Conclusion

Resistance to therapy remains one of the greatest challenges in cancer. Cancer cells can attain resistance through multiple mechanisms, either through selective pressures that allow outgrowth of fit cells, *de novo* alterations in a cell’s physiology, or through plastic adaptations that promote survival under environmental stressors and revert upon resolution of the stress. Mitochondrial adaptations to cancer therapy have gained interest in recent years, primarily for the potential for therapeutic intervention by targeted therapies. Understanding the mechanisms driving mitochondrial metabolism adaptations will be critical in optimizing effective combination therapies, scheduling, and dosages. In addition, differentiating between mitochondrial response adaptations (either permanent or plastic) will be necessary to take advantage of plastic therapeutic windows in the surviving residual disease.

Increased oxphos in cells surviving therapy has been documented in a variety of cancers. Therefore, understanding the energetic sources fueling oxphos is essential for developing targeted therapies. Herein we reviewed evidence that drug resistant cancers rely on fatty acids, glucose, and/or glutamine to feed the TCA cycle and oxphos, and by using drugs to selectively target these pathways, there has been success killing tumor cells in many preclinical studies. Additionally, targeting TCA cycle enzymes or proteins involved in ROS buffering have also proven promising to mitigate drug induced oxphos reliance. While we focused on pathways promoting oxphos adaptations, it is important to consider that other metabolic pathways, such as nucleotide metabolism and the PPP, could be contributing to cancer resistance and metabolic reprogramming (Catanzaro et al., 2015; Brown et al., 2017; Xu et al., 2018; Cao et al., 2019; Pranzini et al., 2022).

An important feature of metabolic rewiring is that it is highly context dependent, often with specificity to tumor and therapy type. These studies demonstrate the critical importance of studying metabolic rewiring so that we can devise therapeutic schemes to overcome the specific type of rewiring that occurs. To address this, more systematic head-to-head studies must be done. For example, comparing various treatments within the same tumor type or expanding studies to include multiple tumor types may provide clarity on what predisposes tumors to mitochondrial metabolic rewiring. It will also be beneficial to move towards a standardization of metabolic profiling and metabolic function assays to aid in our ability to compare data across studies.

In general, there has been a lack of oxphos targeting drugs that have successfully received FDA approval. Metformin is a popular drug used to target oxphos throughout these studies, and while it is FDA approved for management of diabetes, it is known for having many off-target effects. Toxicities with more selective drugs, such as IACS-010759, have also prevented FDA approval. Therefore, research involving oxphos adaptations in cancer may benefit from repurposing already approved drugs which historically are used for other diseases, such as stains for control of cholesterol. A major limitation in this field currently is a lack of animal and human experimental studies that validate *in vitro* and *in vivo* cell line and PDX work. While there is human data which suggests oxphos may be a driving force behind tumor progression, these studies have not investigated this in the context of drug resistance (Hensley et al., 2016; Faubert et al., 2017). Further, clinical trials testing plastic metabolic resistance is limited. In castration resistant prostate cancer, a clinical trial rechallenged patients with abiraterone at a lower dose once prostate specific antigen levels returned to baseline rather than continuous treatment at a higher dose (Gallaher et al., 2018). Patients survived longer under the abridged treatment, demonstrating that leveraging plastic adaptations to therapy may have utility in humans, however, whether metabolic adaptations to abiraterone contributed to resistance was not investigated (Gatenby et al., 2009; Gallaher et al., 2018). Additionally, longitudinal studies utilizing GEMM and PDX mouse models that attain resistance to therapy through mitochondrial rewiring are limited (Fox et al., 2020; van Gastel et al., 2020). Therefore, future studies should consider including human and mouse specimens to validate cell line findings.

Many studies in this review test the efficacy of oxphos targeting drugs either in combination with the standard-of-care drug or in cells already resistant to the therapy of interest. There have been few studies that test oxphos targeting drugs in a true residual setting wherein treatment naive cells are treated first with standard-of-care and then followed up with an oxphos inhibitor once the metabolic reprogramming has occurred. Future studies may benefit from experiments designed in this manner given that it has clinical utility in addressing alternative treatment options for cancers that do not regress with standard-of-care treatment. Further, experiments that utilize this model may have the ability to assess the plasticity of drug induced oxphos adaptations. Most of the studies in this review do not assess the temporal nature, if any, of oxphos rewiring. Extending studies to allow for tumor relapse may reveal the plastic nature of oxphos reliance, suggesting that the timing of oxphos inhibitors may be of critical consideration. Ultimately, understanding the temporal mechanisms of mitochondrial driven adaptations to cancer therapy will lead to better overall care for cancer patients.
